# Serum Pepsinogen as a Biomarker of Gastrointestinal Stromal Tumors (GIST) in Stomach

**DOI:** 10.1002/cam4.71186

**Published:** 2025-09-03

**Authors:** Zhiying Gao, Laizhi Luo, Yueting Han, Yan Sun, Yonghong Huang, Shixia Li, Xingyun Chen, Huimin Yang, Zhijuan Peng, Xinyi Wang, Wei Zhao, Xi Wu, Huan Wu, Jing Bai, Wu Sun, Likun Zhou, Yi Ba

**Affiliations:** ^1^ Peking Union Medical College Hospital Chinese Academy of Medical Sciences & Peking Union Medical College Beijing China; ^2^ Guangzhou Medical University Guangzhou China; ^3^ Key Laboratory of Cancer Prevention and Therapy, National Clinical Research Center for Cancer Tianjin's Clinical Research Center for Cancer, Tianjin Medical University Cancer Institute and Hospital Tianjin China; ^4^ Institute of Pathology Qiqihar Medical University Qiqihar China; ^5^ Handan Central Hospital Handan China; ^6^ People's Hospital of Xiangxi Autonomous Prefecture Xiangxi China; ^7^ Gastrointestinal Department of Tianjin Medical University General Hospital Tianjin China; ^8^ National Cancer Center/National Clinical Research Center for Cancer/Cancer Hospital Chinese Academy of Medical Sciences and Peking Union Medical College Beijing China; ^9^ Ultrasound Department of Sun Yat‐Sen Memorial Hospital Sun Yat‐Sen University Guangzhou China; ^10^ Beijing Friendship Hospital Capital Medical University Beijing China; ^11^ The Comprehensive Cancer Centre of Drum Tower Hospital Medical School of Nanjing University and Clinical Cancer Institute of Nanjing University Nanjing China

**Keywords:** biomarker, gastric atrophy, GIST, serum pepsinogen, stomach

## Abstract

**Background:**

Gastric GISTs (GG) are significant mesenchymal tumors. No biomarker has been identified for GG detection. We first observed mucosal atrophy surrounding GG tumors, leading to the hypothesis that localized atrophy may alter serum pepsinogen (PG) levels. Therefore, we developed a machine learning (ML) model incorporating serum PG levels and clinical features to predict GG and differentiate it from gastric cancer (GC).

**Methods:**

We retrospectively analyzed GG and GC patients with tested PG levels before medical intervention. Seven ML algorithms were assessed, and feature importance was determined using SHapley Additive exPlanations (SHAP). Gastric atrophy was assessed histologically using the updated Sydney System.

**Results:**

After screening 562 GG and 1090 GC patients, 100 GG and 174 GC samples were included. The multilayer perceptron (MLP) model achieved the highest AUC. The final MLP model, which included 4 features—gender, PGI levels, PGI/PGII ratio, and CEA—predicted GG with an AUC of 0.854. Considering clinical practice and the feature importance identified by the final MLP model, we established a Positive‐Gastric‐GIST‐PG‐CEA criterion (PGI < 70 ng/mL, PGI/PGII ratio ≥ 3.0, and CEA ≤ 5 μg/L) referring to the cutoff values revealed by the ROC curve. The Positive‐Gastric‐GIST‐PG‐CEA displayed exceptional performance in predicting GG (AUC = 0.772, accuracy = 0.748, specificity = 0.787, sensitivity = 0.680), with performance comparable to the final MLP model (ΔAUC = 0.082, *p* > 0.05). The contributions of PGI levels, PGI/PGII ratio, and CEA in the Positive‐Gastric‐GIST‐PG‐CEA model performance were 0.33, 0.15, and 0.13 based on SHAP analysis. Histopathological evaluation of gastric mucosal atrophy in 50 GG patients revealed peri‐tumoral glandular atrophy in 29 cases (58%).

**Conclusions:**

The Positive‐Gastric‐GIST‐PG‐CEA criterion is valuable for detecting GG and distinguishing it from GC. Integrating our criteria into existing PG tests could help in GG detection without additional economic expense.

## 1 Introduction

Gastrointestinal stromal tumors (GISTs), which have an annual incidence of 10–15 cases per million population, represent the most common mesenchymal tumors of the gastrointestinal tract and account for 5.7% of all sarcomas [1, 2]. Most GISTs (50%–70%) arise in the stomach. With similar ultrastructure to interstitial pacemaker cells of Cajal, GISTs are recognized as predominantly associated with KIT or PDGFRA [3, 4, 5]. Immunohistochemistry (IHC) of KIT, discovered on GIST‐1 (DOG‐1) and CD34, has been proven to be reliable in GIST diagnosis [6, 7, 8, 9]. The incidence of clinical GIST sharply increased to 10–22 per million per year worldwide with the improvement of pathological understanding and the wide use of CD117 IHC staining since 2000 [10, 11, 12, 13, 14, 15]. However, the true incidence is much higher than that if pathological microscopic GISTs (< 1 cm) and minimal gastric GISTs (GG) (< 2 cm) are included [16, 17, 18, 19]. Complete resection avoiding tumor rupture is the mainstay strategy used to cure GISTs. Tyrosine kinase inhibitors (TKIs) increased the 5‐year survival of GG from 46% to 66%–90% [14, 20, 21, 22, 23, 24, 25]. However, most advanced stages, which account for approximately 20% of GG at diagnosis, are still incurable [20, 26]. Because most GG (58%) are asymptomatic, they are hardly alert to medical consultation [26, 27]. For GG < 5 cm, which have a better prognosis than larger size GG, 65% of patients were asymptomatic [27]. Notably, symptomatic presentation independently correlates with poorer outcomes and reduced 5‐year disease‐specific survival compared to incidentally detected cases [28]. Despite significant advances in imaging technologies including CT and magnetic resonance imaging (MRI), misdiagnoses still occur. This underscores the urgent need for noninvasive, radiation‐free serum biomarkers to enable detection of GG and improve patient outcomes.

Pepsinogen (PG) is the precursor of pepsin specifically produced in the stomach. PGII is synthesized and secreted by chief cells in both gastric oxyntic glands at the corpus and pyloric glands in the gastric antrum, whereas PGI is secreted by only the corpus stomach mucosa. Although only 1% of PG is secreted into the bloodstream, the serum/plasma PG levels are quite stable for approximately 10 years in more than 90% of adults [29]. This high stability makes serum PG levels a good noninvasive biomarker for oxyntic gland population decrease in the stomach. It increases in the early stage of inflammation, *Helicobacter pylori* (*Hp*) infection, and gastric ulcers, but declines with progressive mucosal atrophy. A decrease in the oxyntic gland population leads to a reduction in serum PGI, which may result in a lower PGI/PGII ratio. Serum PGI < 70 ng/mL plus PGI/PGII ratio < 3 is a widely used criterion for screening atrophic gastritis (AG).

In GG hematoxylin and eosin‐stained (H&E) slides, we found that some glands surrounding GG are atrophic, while no atrophy occurred in the glands of the resection margin. Because PGI was highly related to gastric mucosa atrophy, we hypothesize that serum PGI has a unique expression model in GG. To test our hypothesis, we first searched the data from June 1, 2012, to December 31, 2012, in the Tianjin Cancer Hospital database. Patients who met the two criteria were included. First, GG was diagnosed by both H&E and CD117 positivity. Second, serum PGI and PGII levels were measured prior to medical intervention. Ten patients fulfilled the criteria. The pathological slides of six patients were available. The slides of two patients had no surrounding gastric mucosa. Three of the remaining four patients showed obvious mucosal atrophy (Table S1). The serum PGI levels in four patients were abnormal, with values < 70 ng/mL.

To further validate our hypothesis, we conducted a retrospective cohort study including GG and gastric cancer (GC). The rationale for selecting GC as controls is that GC and GG represent the most common epithelial malignancy and mesenchymal tumor of the stomach, respectively. They constitute the predominant malignant gastric tumors where current non‐serological diagnostic methods lack discriminatory power, and conventional imaging carries inherent misclassification risks. A noninvasive biomarker would not only facilitate GG screening but also provide additional diagnostic clues beyond imaging for pathologically unconfirmed gastric tumors. Through systematic comparison of serological profiles and clinicopathological characteristics between GC and GG patients, we developed and validated a clinically interpretable machine learning (ML) model capable of both predicting GG and distinguishing it from GC.

## 2 Methods

### 2.1 Study Patients

We retrospectively screened GG inpatients and outpatients in our hospital from January 1, 2013 to April 30, 2019. GC patients were also screened from a database (those patients were tested for PG levels before medical intervention and confirmed to be diagnosed with adenocarcinoma at our hospital from July 2012 to August 2016). The inclusion criteria were as follows: (1) Patients of any age or sex diagnosed with GG or gastric adenocarcinoma and (2) patients with tested PG levels before medical intervention. The diagnosis of GIST was based on morphological and IHC findings. Positive CD117 and/or DOG1 and/or CD34 were determined by IHC. The exclusion criteria were as follows: (1) Patients with renal failure or synchronous other GI cancer; (2) patients with previous gastric surgery; (3) patients with previous blood transfusion within 4 weeks before the PG test; (4) patients with a history of *Hp* eradication; (5) patients who took proton pump inhibitors (PPIs), nonsteroidal anti‐inflammatory drugs (NSAIDs), or traditional Chinese medicine (TCM) within 4 weeks before the PG test; and (6) patients with a gastric ulcer history. Serum PG and carcinoembryonic antigen (CEA) levels were extracted from medical records. This retrospective study was approved by the Institutional Review Board of Tianjin Cancer Hospital. The requirement for informed consent was waived because this was a retrospective study, and all patients were discharged. The authors vouch for the accuracy of the data. No commercial entity was involved.

### 2.2 Patient Enrollment

We first reviewed the medical records to confirm the diagnosis and drug consumption of the patients for further assessment. Then, all participants or their family members were interviewed by telephone about their drug consumption and blood transfusion history before the PG test.

### 2.3 Sample Collection and Analysis

Following the handbook of Tianjin Cancer Hospital, overnight fasting serum levels of PGI and PGII were measured using the chemiluminescence microparticle immunoassay method (ARCHITECT PGI and PGII reagent kit, Abbott, US) in accordance with the instructions of the manufacturer. CEA was measured by Elecsys CEA kit (Roche Diagnostics GmbH, Germany). Detection of *Hp* infection in patients was performed using the 13C‐urea breath test or antibody‐based assays.

### 2.4 Development and Validation of Machine Learning Model

Prior to model development, uniform preprocessing was applied across the training, validation, and test sets. Variables with > 20% missing values were excluded, and residual missing data were imputed using missForest, a random forest–based algorithm robust to non‐linearity and complex data structures. To ensure rigorous evaluation, the cohort (comprising GG and GC patients) was partitioned into a 70% training set and a 30% heldout test set for internal validation, mitigating overfitting risks.

Feature importance was quantified using SHAP (SHapley Additive exPlanations), which leverages game theory to interpret model outputs. To optimize predictive efficiency, features were incrementally incorporated into the model in descending order of SHAP‐derived importance. This stepwise approach allowed systematic evaluation of the contribution of each variable while maintaining parsimony.

We employed seven widely used ML algorithms for assisted diagnosis prediction, including: logistic regression (LR) [30], random forest (RF) [31], extreme gradient boosting (XGBoost) [32], light gradient boosting machine [LightGBM] [33], multilayer perceptron (MLP) [33], support vector machine (SVM) [34], and naive Bayes (NB) [35]. The LR model served as an interpretable baseline for establishing linear feature relationships. Ensemble methods (RF, XGBoost, and LightGBM) were selected for their demonstrated robustness in handling heterogeneous features and resistance to overfitting. SVM and MLP were included to address potential nonlinear separability in the data, while NB provided computational efficiency for probabilistic inference.

A total of 10 GG‐related features were selected for the development of the prediction models. The reliability of the models was assessed using commonly applied evaluation metrics, including the area under the receiver operating characteristic curve (AUC‐ROC), sensitivity, specificity, accuracy, positive predictive value (PPV), negative predictive value (NPV), and F1‐score. For model training, a 5‐fold cross‐validation approach was employed. To enhance model performance, the SHAP method was used to identify clinically significant features contributing to the predictions. By removing irrelevant or noisy features, SHAP helps mitigate collinearity issues, thereby improving model robustness.

### 2.5 Model Interpretation

To address the “black box” nature of ML models, we employed the SHAP method to evaluate feature importance and interpret the predictions of the models. In this study, beyond interpreting feature contributions, SHAP values were also used to assess the clinical relevance of the predictive models [36]. For model interpretation, we applied globally explainable methods [37]. This global explanation approach provides consistent and reliable attribution values for each feature, shedding light on the relationships between input variables and GG outcomes.

### 2.6 Histopathological Assessment

Hematoxylin–eosin (H&E) and IHC slides were reviewed by two pathologists for atrophy evaluation. If there were disagreements between them, a third pathologist judged whether there was atrophy. Because there were clear boundaries between the tumor lesions and surrounding glands in GG rather than in GC, only GG patients were evaluated for gland atrophy. Blinding was not applied to the pathologists because it is easy to recognize GG on H&E‐stained slides. Gastric mucosal atrophy was assessed based on the updated Sydney System (USS) [38]. All specimens were stained with H&E. Glandular atrophy was graded using a visual analogue scale (0: none, 1: mild, 2: moderate, 3: severe) according to the USS criteria. The kappa value and its 95% CI were calculated to describe the agreement of two pathologists for adjacent gastric mucosa atrophy.

### 2.7 Statistical Analysis

Continuous variables were reported as medians and compared using the *t* test for two groups. Categorical variables were expressed as frequencies (percentages) and analyzed using Pearson's chi‐square test or Fisher's exact test, as appropriate. All statistical tests were two‐tailed, with a *p* < 0.05 considered statistically significant. Analyses were performed using Python 3.9 (https://www.python.org) and SPSS (v23.0; IBM Corp., Armonk, NY, USA).

## 3 Results

### 3.1 Patients Characteristics

**FIGURE 1:**
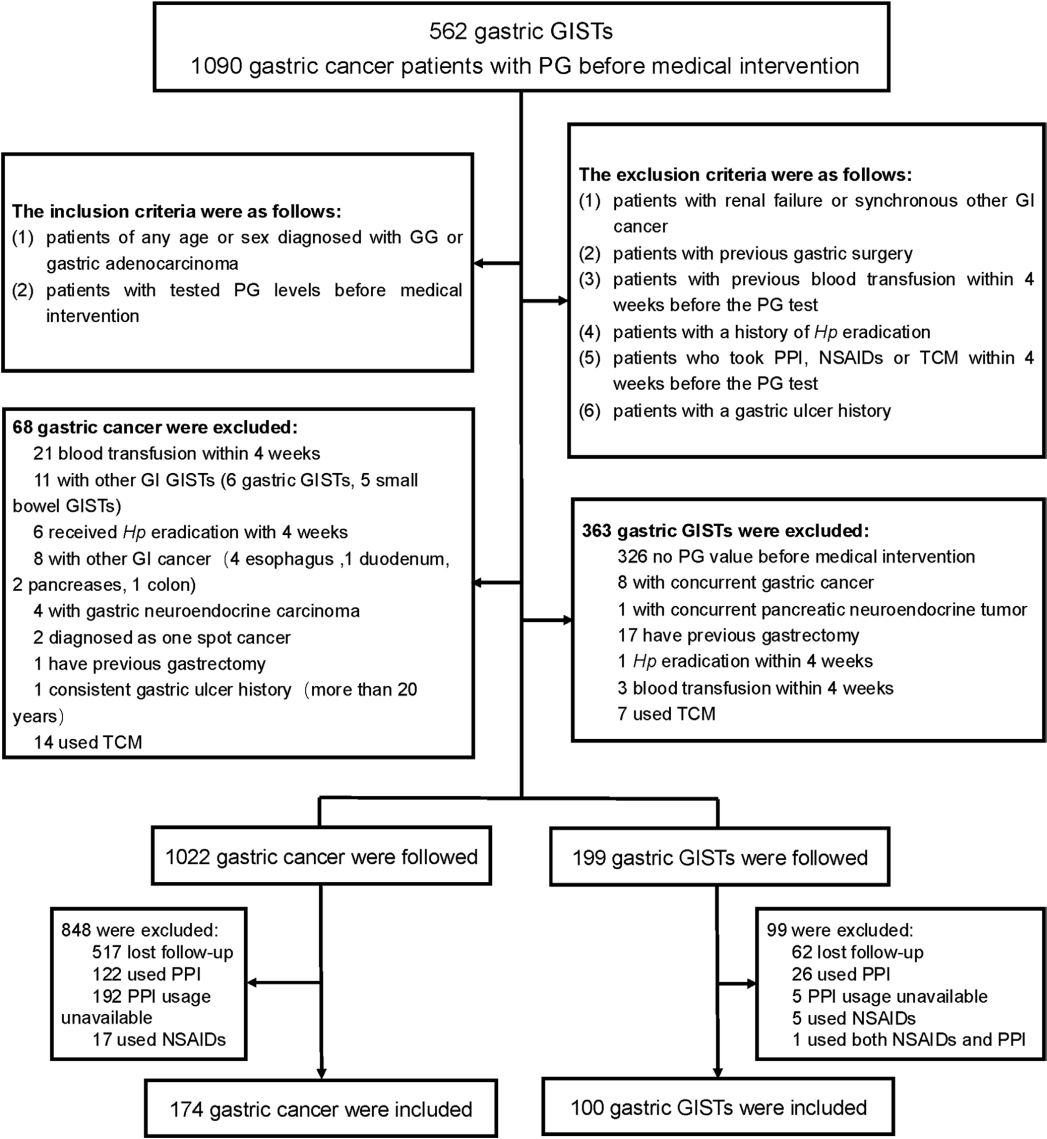
Study flowchart. GI, gastrointestinal; GISTs, gastrointestinal stromal tumors; *Hp*, *Helicobacter pylori*; NSAIDs, nonsteroidal anti‐inflammatory drugs; PG, pepsinogen; PPIs, proton pump inhibitors; TCM, traditional Chinese medicine.

From January 1, 2013 to April 30, 2019, a total of 562 GG patients and 1090 GC patients were screened. After evaluation, 199 GG patients and 1022 GC patients were interviewed by telephone. In addition to loss to follow‐up, PPI usage was the main reason for exclusion (27 GG patients and 122 GC patients). Finally, 100 GG patients and 174 GC patients were included (Figure 1). The median age of all included patients was 60 (range 27–83) years. The detailed clinicopathological data are shown in Table 1.

**TABLE 1:** Demographic and clinical characteristics of the patients.

	Gastric GIST (*N* = 100)	Gastric cancer (*N* = 174)	*p*
Age, median (range)—years	59.5 (27.0–82.0)	61.0 (28.0–83.0)	0.134
Male sex—no. (%)	32 (32)	126 (72)	**< 0.001**
Alcohol drinking—no. (%)			
Noncurrent drinker	72 (77.4)	112 (67.1)	0.79
Current drinker	21 (22.6)	55 (32.9)	
Unknown	7 (7.0)	7 (4.0)	
Smoking—no. (%)			
Noncurrent smoker	68 (73.1)	116 (69.0)	0.49
Current smoker	25 (26.9)	52 (31.0)	
Unknown	7 (7.0)	6 (3.4)	
Tumor size, median (range)—cm	5.0 (0.5–23.0)	5.0 (0.8–19.0)	0.067
Tumor resection—no. (%)			
No	4 (4.0)	52 (30.0)	**< 0.001**
Yes	96 (96.0)	122 (70.0)	
Operative type			
Laparotomy—no. (%)	56 (56.0)	103 (59.2)	**< 0.001**
Laparoscopic surgery—no. (%)	28 (28.0)	2 (1.1)	
ESD or EFR—no. (%)	12 (12.0)	0 (0)	
Palliative resection—no. (%)	0 (0)	15 (8.6)	
No surgery—no. (%)	4 (4.0)	54 (31.1)	
Metastasis—no. (%)			
No	94 (94.0)	102 (58.6)	**< 0.001**
Yes	6 (6.0)	43 (24.7)	
Unknown	0 (0)	29 (16.7)	
Tumor location—no. (%)			
EGJ	43 (43.0)	36 (20.7)	**< 0.001**
Corpus	47 (47.0)	59 (33.9)	
Corner or antrum	9 (9.0)	61 (35.1)	
Whole stomach	0 (0)	6 (3.4)	
Unknown	1 (1.0)	12 (6.9)	

*Note:* Bold values indicate *p* < 0.05, which is considered statistically significant.

Abbreviations: EFR, endoscopic full thickness resection; EGJ, esophagogastric junction; ESD, endoscopic submucosal dissection;.

As the clinical characteristics of GG and GC are different in the real world, several baseline factors were imbalanced between the two groups. Most GG patients were located at the esophagogastric junction (EGJ), cardia, fundus, and corpus (90/100) compared with 55% (95/174) of GC (*p* < 0.05). The GC group enrolled more males (72% vs. 32%, *p* < 0.05). More GC patients had distant metastasis, and fewer GC patients underwent radical surgery. Other factors, including age, alcohol consumption, smoking, and tumor size (median size, 5.0 cm vs. 5.0 cm), were not significantly different between the two groups.

The positive rates were 95%, 98% and 98% for CD117, DOG‐1, and CD34, respectively, in the GG group. Most patients had C‐kit exon 11 mutations (41 out of 56 patients who had tested C‐kit mutations). Forty‐eight percent of GG patients were asymptomatic (Table S2).

### 3.2 Binary Logistic Regression Analysis of Risk Scores in Patients With GG and GC Subjects

Multivariate analysis identified significant discriminators between GG and GC (Table 2). The risk of GG was significantly higher than GC in females (OR = 9.31, 95% CI: 3.09–28.09, *p* < 0.001). In contrast, patients with tumors located at the corner and antrum exhibited a lower risk of GG (OR = 0.15, 95% CI: 0.05–0.49, *p* = 0.002). Serum biomarker analysis revealed an inverse relationship between elevated PGI levels and GG (OR = 0.982, 95% CI: 0.972–0.992, *p* < 0.001). Additionally, a higher PGI/PGII ratio was associated with an increased risk of GG (OR = 1.422, 95% CI: 1.172–1.726, *p* < 0.001). Moreover, elevated CEA levels were correlated with a reduced probability of GG (OR = 0.774, 95% CI: 0.614–0.976, *p* = 0.030). No significant associations were observed for age, smoking, alcohol history, metastasis, or tumor size. These findings underscore the importance of distinct clinicopathological and serological predictors of GG, providing valuable insights for risk stratification and diagnostic evaluation.

**TABLE 2:** Multivariate logistic regression analysis for the GG risk factors.

Variables	Multivariable model		
	β	OR (96% CI)	*p*
Gender			
Male	—		
Female	2.232	9.314 (3.089–28.089)	**< 0.001**
Age, years	−0.008	0.992 (0.955–1.03)	0.667
Smoking status			
Noncurrent smoker	—		
Current smoker	0.785	2.192 (0.523–9.179)	0.283
History of alcohol			
Noncurrent drinker	—		
Current drinker	0.029	1.030 (0.245–4.334)	0.968
Tumor location			
EGJ	—		
Corpus	−0.593	0.553 (0.209–1.459)	0.231
Corner or antrum	−1.903	0.149 (0.045–0.494)	**0.002**
Tumor metastasis			
Yes	—		
No	−0.888	0.411 (0.076–2.226)	0.302
Tumor size	0.096	1.101 (0.958–1.264)	0.175
PGI (ng/mL)	−0.019	0.982 (0.972–0.992)	**< 0.001**
PGI/PGII ratio	0.352	1.422 (1.172–1.726)	**< 0.001**
CEA (μg/L)	−0.256	0.774 (0.614–0.976)	**0.030**

*Note:* Bold values indicate *p* < 0.05, which is considered statistically significant.

Abbreviations: CEA, carcinoembryonic antigen; EGJ, esophagogastric junction; PGI, pepsinogen I; PGII, pepsinogen II;.

### 3.3 Model Development and Performance Comparison

Following assessing the risk factors, we developed and compared seven distinct ML models (RF, LR, LightGBM, MLP, XGBoost, SVM, and NB) to predict GG. Model performance was evaluated using standard metrics including AUC, accuracy, sensitivity, specificity, PPV, NPV, and F1‐score. As detailed in Table 3, the MLP model (AUC = 0.865, 95% CI: 0.781–0.930) demonstrated the highest predictive ability for GG in the seven ML models. This neural network architecture consistently outperformed other models across multiple evaluation metrics, highlighting its optimal suitability for GG risk stratification.

**TABLE 3:** The discriminative performances of these seven models.

Model	AUC (95% CI)	Accuracy (95% CI)	Specificity (95% CI)	Sensitivity (95% CI)	PPV (95% CI)	NPV (95% CI)	F1‐score (95% CI)
MLP	0.865 (0.781, 0.930)	0.783 (0.586, 0.868)	0.811 (0.696, 0.917)	0.733 (0.565, 0.875)	0.688 (0.516, 0.853)	0.843 (0.516, 0.853)	0.710 (0.556, 0.825)
RF	0.863 (0.760, 0.938)	0.795 (0.371, 0.867)	0.943 (0.868, 1.000)	0.533 (0.333, 0.704)	0.842 (0.650, 1.000)	0.781 (0.650, 1.000)	0.653 (0.457, 0.792)
LR	0.861 (0.776, 0.93)	0.735 (0.483, 0.819)	0.792 (0.678, 0.896)	0.633 (0.450, 0.794)	0.633 (0.444, 0.815)	0.792 (0.444, 0.815)	0.633 (0.464, 0.764)
LGB	0.861 (0.768, 0.935)	0.783 (0.480, 0.855)	0.868 (0.774, 0.944)	0.633 (0.448, 0.800)	0.731 (0.545, 0.889)	0.807 (0.545, 0.889)	0.679 (0.513, 0.812)
XGBoost	0.852 (0.756, 0.930)	0.771 (0.370, 0.843)	0.906 (0.82, 0.978)	0.533 (0.346, 0.714)	0.762 (0.560, 0.933)	0.774 (0.560, 0.933)	0.627 (0.439, 0.769)
SVM	0.845 (0.757, 0.920)	0.747 (0.483, 0.831)	0.811 (0.692, 0.915)	0.633 (0.450, 0.794)	0.655 (0.467, 0.833)	0.796 (0.467, 0.833)	0.644 (0.476, 0.778)
NB	0.838 (0.739, 0.915)	0.687 (0.590, 0.974)	0.566 (0.424, 0.691)	0.900 (0.769, 1.000)	0.540 (0.386, 0.686)	0.909 (0.386, 0.686)	0.675 (0.533, 0.787)

Abbreviations: AUC, area under the curve; LightGBM, light gradient boosting; LR, logistic regression; MLP, multilayer perceptron; NB, naive Bayes; NPV, negative predictive value; PPV, positive predictive value; RF, random forest; SVM, support vector machine; XGB, extreme gradient boosting.

### 3.4 Identification of the Final Model

**FIGURE 2:**
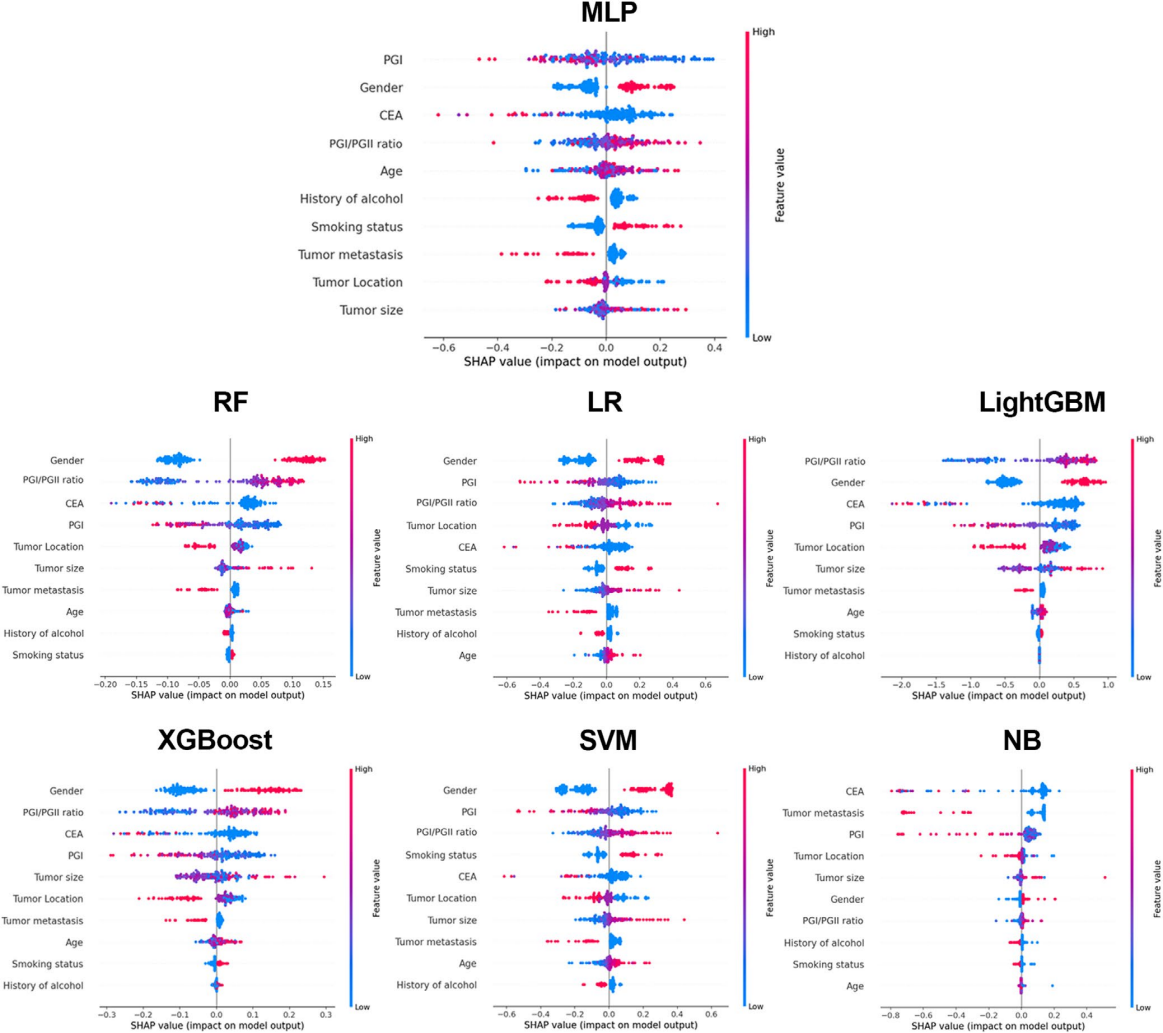
The SHAP summary plots of the 10 features for the 7 ML models. LightGBM, light gradient boosting machine; LR, logistic regression; MLP, multilayer perceptron; NB, naive Bayes; RF, random forest; SVM, support vector machine; XGBoost, extreme gradient boosting.

**FIGURE 3:**
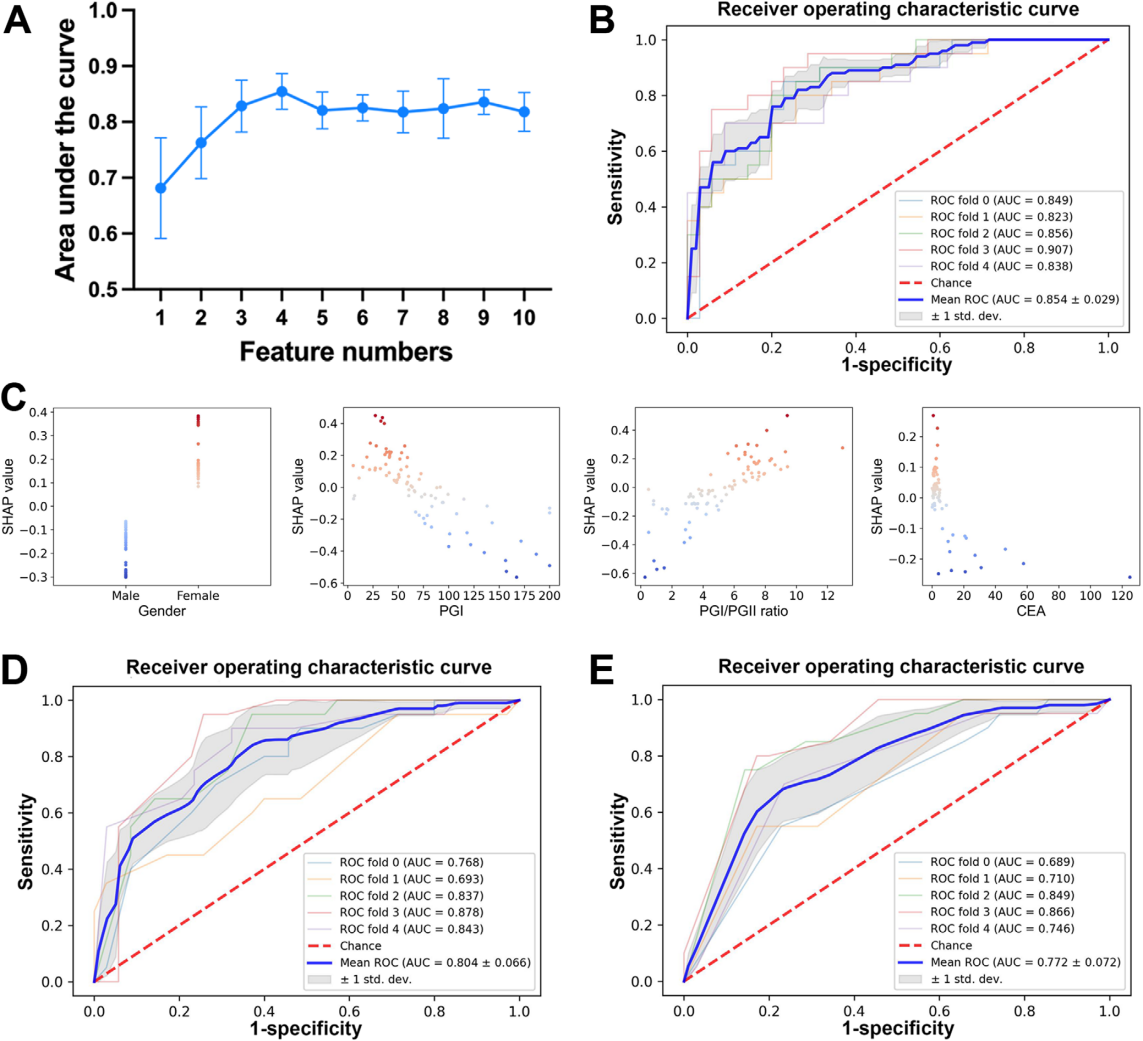
Development and interpretation of the final MLP model using SHAP analysis. (A) MLP model AUC value changes with different numbers of features. (B) The 5‐fold cross‐validation AUC of the final MLP model. (C) SHAP dependence plot of the top 4 features for the MLP model. (D, E) The 5‐fold cross‐validation performance of serological panels: (D) PGI < 70 ng/mL + PGI/PGII ≥ 3.0 + CEA ≤ 5 μg/L + gender; (E) PGI < 70 ng/mL + PGI/PGII ≥ 3.0 + CEA ≤ 5 μg/L. AUC, area under the curve; MLP, multilayer perceptron; ROC, receiver operating characteristic.

To systematically evaluate the impact of feature selection on model performance, we employed SHAP analysis to identify the 10 features in the seven ML models. Figure 2 displays the SHAP summary plots analyzing the contribution of the 10 features across the seven ML models. Notably, the top 4 most influential features were gender, PGI levels, PGI/PGII ratio, and CEA in most models. Additionally, we incrementally added features to the model training process based on their SHAP‐derived importance rankings. Through this stepwise feature reduction approach, we identified the optimal feature subset for the final MLP model. As demonstrated in Figure 3A, the simplified 4‐feature model (gender, PGI, PGI/PGII ratio, and CEA) achieved the highest AUC compared to the other models. This supports the utility of the reduced model as a parsimonious yet effective alternative for clinical application.

The model development process involved 100 GG patients and 174 GC patients. After performing 5‐fold cross‐validation, the MLP model, which included four features (gender, PGI, PGI/PGII ratio, and CEA, Gender‐Positive‐Gastric‐GIST‐PG‐CEA), achieved an AUC of 0.854, with an accuracy of 0.792, specificity of 0.867, sensitivity of 0.660, PPV of 0.754, NPV of 0.820, and an F1 score of 0.693 in predicting GG (Figure 3B, Table S3). The SHAP dependence plot provided valuable insights into the influence of individual features on the prediction model. The true values versus SHAP values of these four features are shown in Figure 3C, with SHAP values greater than zero indicating an elevated risk of GG. Furthermore, ROC curve analysis revealed that the cutoff values for GG were PGI 70.85 ng/mL, PGI/PGII ratio 3.996, and CEA 4.16 μg/L, which are close to the clinically established thresholds for PGI (70 ng/mL), PGI/PGII ratio (3.0), and CEA (5 μg/L), respectively.

Considering clinical practicality and risk scores, we established clinical threshold values (PGI < 70 ng/mL, PGI/PGII ratio ≥ 3.0, CEA ≤ 5 μg/L) to create a new binary variable combined with gender, ultimately developing the Gender‐Positive‐Gastric‐GIST‐PG‐CEA model. This model achieved an AUC of 0.804 in predicting GG (Figure 3D, Table S3). Although the model demonstrated relatively high specificity (0.908), its sensitivity was comparatively low (0.500), which may increase the risk of false negatives in clinical applications. To optimize its performance, we reevaluated the model using only PGI < 70 ng/mL, PGI/PGII ratio ≥ 3.0, and CEA ≤ 5 μg/L (Positive‐Gastric‐GIST‐PG‐CEA) through 5‐fold cross‐validation. The final Positive‐Gastric‐GIST‐PG‐CEA model achieved an AUC of 0.772, accuracy of 0.748, specificity of 0.787, sensitivity of 0.680, PPV of 0.649, NPV of 0.811, and an F1 score of 0.663 in predicting GG (Figure 3E, Table S3). These results indicate that the simplified model based on clinical experience thresholds achieved comparable diagnostic performance to the ML‐optimized model (ΔAUC = 0.082; *p* > 0.05), further validating the fundamental value of established clinical standards in GG screening.

### 3.5 Feature Importance and Interpretability Analysis

**FIGURE 4:**
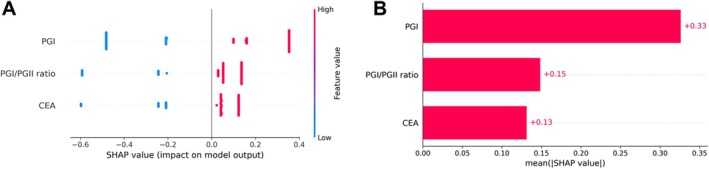
Global interpretation of the final model using SHAP analysis. (A) The SHAP summary dot plot. The color gradient indicates feature values (red for high, blue for low), and the horizontal SHAP value reflects the feature's influence on GG prediction. Feature categorization: PGI < 70 ng/mL, PGI/PGII ratio ≥ 3.0, or CEA ≤ 5 μg/L = 1; others = 0. (B) The SHAP summary bar plot.

Given the importance of interpretability for clinician acceptance, the SHAP method was employed to interpret the final model, which is based on PGI < 70 ng/mL, PGI/PGII ratio ≥ 3.0, and CEA ≤ 5 μg/L for GG prediction. The global explanation analysis provided insights into the model's overall functionality, with SHAP values visualized in Figure 4A. The SHAP summary bar plot ranks the features by their mean absolute SHAP values, highlighting their relative importance in the model's decision‐making process. The quantitative assessment of feature contributions showed that the contributions of PGI, PGI/PGII ratio, and CEA were 0.33, 0.15, and 0.13, respectively (Figure 4B).

### 3.6 Peri‐Tumoral Mucosal Atrophy in GG Patients

**FIGURE 5:**
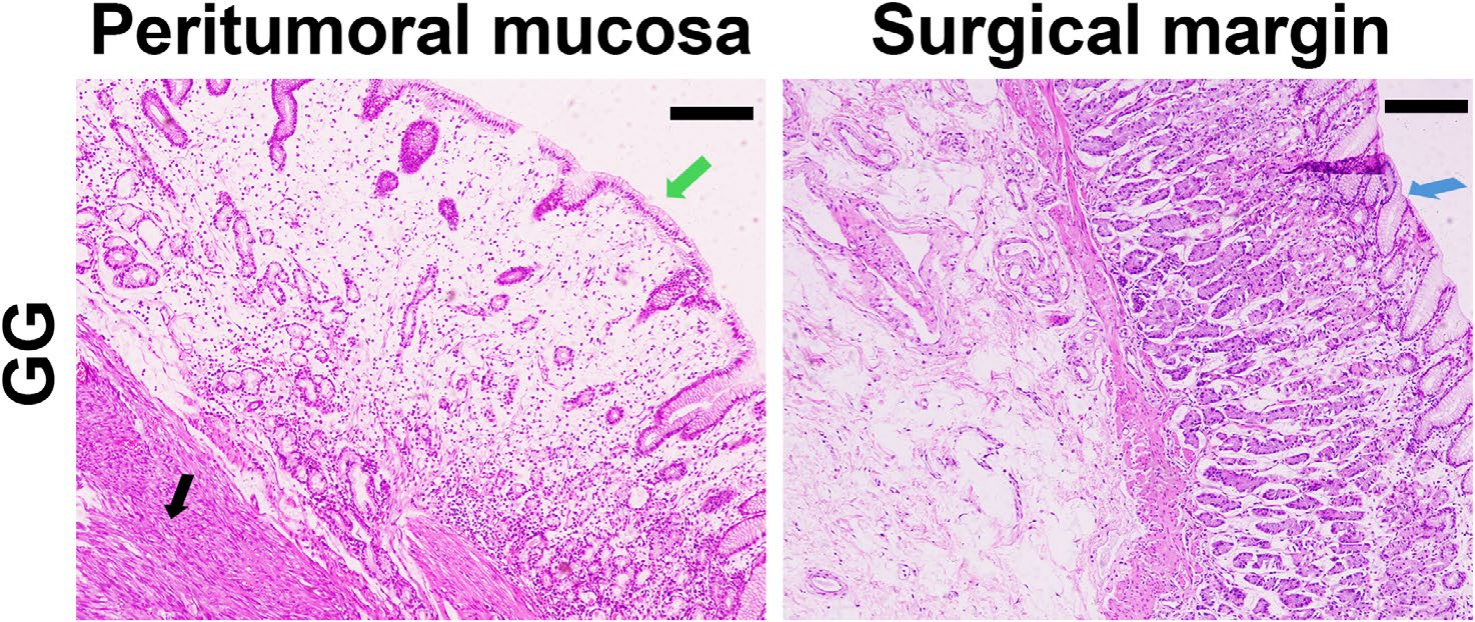
H&E‐stained peritumoral mucosa and margin in GG. Atrophic glands (green arrows), normal glands (blue arrows), and GG tumor cells (black arrows) are indicated. Scale bar: 100 μm.

Histologically evaluable H&E‐stained sections, encompassing both the tumor and adjacent gastric mucosa, were obtained from 50 GG patients (7 patients did not have slides; 42 slides did not include the surrounding mucosa and 1 slide was post‐neoadjuvant specimen). Using the USS scoring system, we identified peri‐tumoral mucosal atrophy in 29 cases (58%), with excellent inter‐rater reliability (*κ* = 0.88, 95% CI: 0.74–1.0). The USS scores for peri‐tumoral mucosa in GG patients are detailed in Table S4. The atrophy was predominantly mild‐to‐moderate (24/29, 82.8%; USS scores 1–2), with 5 cases (17.2%) showing severe atrophy (USS score 3). Although significant atrophy was observed in the peri‐tumoral mucosa, all resection margins retained normal mucosal architecture without atrophic changes (Figure 5). Based on the exclusive origin of PG from gastric glands and the observed peri‐tumoral mucosal atrophy in GIST patients, these findings further support our ML‐derived conclusion that alterations in serum PG levels can serve as a potential predictive marker for GG screening.

### 3.7 Subgroup Analysis of Sensitivity

As PG is produced by certain stomach locations and influenced by several factors, it is important to address whether the sensitivity is reproducible in subgroups. Subgroup analysis of the GG group was stratified by tumor location, size, recurrence risk, lesion growth pattern, initial symptoms, sex, and age. The results are listed in Table S5. The sensitivity of the Positive‐Gastric‐GIST‐PG‐CEA was as high as 91% in tumors ≤ 1 cm (10 out of 11 patients) and in patients younger than 50 years (20/22). This criterion also detected 90% zero recurrent risk GG patients (14/17) and all moderate recurrent patients (9/9). The lesion growth pattern of 43 patients was obtained from computed tomography (CT) scan and endoscopy data. The analysis showed that there was no significant difference between exophytic and endophytic masses (20/29 vs. 9/14, *p* > 0.1). Two of four patients who showed no mass but limited thicker gastric wall on CT scan fulfilled the Positive‐Gastric‐GIST‐PG‐CEA. The sensitivities were not different between males and females (*p* > 0.05). Correlations between PGI level and GIST recurrent risk or tumor size were not detected. All three criteria showed higher sensitivities in *Hp* negative patients than in *Hp* positive patients (*p* < 0.05). Correlations between PGI level and GIST recurrent risk or tumor size were not detected.

Subgroup analysis of the GC group was stratified by tumor location, size, grade of tumor differentiation, clinical stage, sex, and age. The results are listed in Table S6. The sensitivity of Positive‐Gastric‐GIST‐PG‐CEA decreased from 38% to 11% with the clinical stages from stage I to stage IV.

## 4 Discussion

Even though GG patients have the best prognosis among all GIST patients, nearly 30% of GG cases eventually relapse or metastasize after curative resection of the primary tumor [39]. The most important recurrence factors are tumor size, tumor rupture, and mitotic rate per 50 high‐power fields. For example, the recurrence rate is 0% for GG patients with lesions ≤ 2 cm [40]. Generally, a smaller GG often means a lower mitotic rate, a lower chance of rupture, a higher chance of receiving less invasive surgery, and a better prognosis with TKI therapy [41]. Early detection has been proven to improve the treatment outcomes of GG in Japan [27]. However, there is still a lack of a reliable noninvasive GG detection method. The reason may be that there are no specific symptoms or GG secreting factors. In fact, 48% of GG patients in our study were asymptomatic. Based on our Positive‐Gastric‐GIST‐PG‐CEA criterion, the serum PG test showed high sensitivities in both asymptomatic GG patients (63%) and small GG patients (14 out of 17 patients with lesions ≤ 2 cm). The benefit of detecting GG ≤ 2 cm is still unclear. However, 11.4% of GISTs < 2 cm were metastatic (regional/distant) [42]. Guidelines recommend different strategies for GG lesions < 2 cm. The National Comprehensive Cancer Network recommended conservative follow‐up for GG < 2 cm without high‐risk features. The European Society for Medical Oncology and Japanese guidelines recommend resection for all GISTs < 2 cm. However, studies found that some GG < 2 cm would enlarge after years of follow‐up [43, 44].

Including a control group of healthy individuals or patients with non‐neoplastic gastric conditions is essential for a new biomarker. However, as serum PG (PGI and PGII) levels combined with *Hp* antibody status have been established for decades as indicators of atrophic gastritis. Normal PGI and PGI/PGII ratio values in healthy subjects are well documented [45, 46]. Most studies and manufacturer guidelines (like the Abbott ARCHITECT kit we used) define PG positivity for atrophic gastritis as PGI < 70 ng/mL and PGI/PGII ratio < 3. Studies with healthy controls report mean PGI levels were 152.95 ± 65.92 ng/mL [47] and 89.24 ± 39.42 ng/mL [48], and studies in endoscopically confirmed non‐atrophic subjects report levels of 155.18 ± 65.69 ng/mL [47] and 107.1 ± 49.5 ng/mL [49]. Another Chinese study showed that only 1 out of 30 endoscopically normal subjects had PGI < 70 ng/mL [50]. Collectively, these data indicate that the normal PGI value is > 70 ng/mL in healthy subjects. Consequently, our “Positive‐Gastric‐GIST‐PG” criterion (PGI < 70 ng/mL and PGI/PGII ratio ≥ 3) can distinguish GG from most healthy controls (PGI > 70 ng/mL). Thus, our study did not select healthy subjects as controls.

In clinical practice, AG is the most common non‐neoplastic condition requiring assessment due to its association with GC. Both PGI levels and PGI/PGII ratios demonstrate progressive decline from normal mucosa to extensive atrophy, independent of *H. pylori* infection status. Although various diagnostic thresholds exist, the combined criteria of PGI < 70 ng/mL and PGI/PGII ratio < 3 have gained widespread acceptance for predicting AG. A meta‐analysis showed that for AG screening, 9 studies used PGI < 70 ng/mL, 8 used PGI/PGII ratio < 3, and 8 used both PGI < 70 ng/mL and PGI/PGII ratio < 3 [51]. Both the Japanese GC Association and American Gastroenterological Association recommend PGI < 70 ng/mL and PGI/PGII ratio < 3 for chronic gastritis detection [52, 53], with sensitivities of 66.7%–84.6% and specificities of 73.5%–87.1% [52]. Since PGI/PGII ratio < 3 indicates AG, our Positive‐Gastric‐GIST‐PG‐CEA criteria (PGI < 70 ng/mL, PGI/PGII ratio ≥ 3.0, and CEA ≤ 5 μg/L) can distinguish GG from AG in most cases.

Although PGI levels < 70 ng/mL combined with PGI/PGII ratios < 3 serve as screening markers for GC, this combination predominantly identifies intestinal‐type GC—the terminal stage in Correa's cascade of precancerous lesions. However, the marked heterogeneity of GC restricts the universal utility of PG‐based screening. Notably, diffuse‐type GC develops through a distinct pathogenetic pathway with greater genetic predisposition [54, 55] and demonstrates lower detection reliability when using atrophy‐associated PG level alterations. By employing GC as a control group, our study directly addresses a crucial clinical gap: as the stomach's two predominant malignancies, GC (the most prevalent epithelial cancer) and GG (the most common mesenchymal neoplasm) currently lack validated serum biomarkers for reliable differentiation. The development of noninvasive biomarkers capable of distinguishing between GC and GG therefore fulfills an important unmet clinical need. Our experimental design, which compares GG with GC, specifically focuses on resolving this clinically essential diagnostic dilemma.

To reduce the biases inherent in the retrospective nature, we considered as many factors as possible. For example, because multiple factors, including race, age, gender, smoking/drinking history, PP use, and *Hp* infection, may influence serum PG levels [52], we excluded patients who received PPIs, NSAIDs, *Hp* eradication therapy, or TCM to minimize the drug impact on PG level. Because most GG patients with gastric bleeding received PPIs as first aid medicine, only 1 patient with gastric bleeding was included in our study. In fact, we also analyzed the impact of PPIs on the PG level of GG patients (data not shown). Only 2/11 and 3/16 patients fulfilled the Positive‐Gastric‐GIST‐PG‐CEA criterion among patients who received PPIs within 24 h and 2 weeks before the PG test, respectively. This suggests that PPIs may significantly compromise the diagnostic efficacy of the Positive‐Gastric‐GIST‐PG‐CEA criterion. Even with these efforts, information biases are inevitable. For instance, *Hp* infection status was confirmed in only 31% of GG patients and 18% of GC patients. Notably, GC patients often show reduced motivation to undergo *Hp* testing after diagnosis. Future studies should determine whether distinct cutoff values should be established for *H. pylori* ‐positive versus *H. pylori* ‐negative groups.

The diagnostic applicability of our Positive‐Gastric‐GIST‐PG‐CEA criterion to non‐gastric GISTs requires further investigation. Tumor location is a recognized independent prognostic factor in GIST. For instance, duodenal GISTs (representing 3%–5% of cases) demonstrate superior survival outcomes compared to GG. This survival advantage likely originates from their anatomical proximity to critical structures—including the pancreatic head, common bile duct, and ampulla of Vater—which predisposes to early symptomatic bleeding. Consequently, these tumors are typically detected earlier, yielding smaller median tumor size (4 cm vs. 6–7 cm in gastric/small bowel GISTs) and lower mitotic activity [28]. In contrast, GG generally exhibits indolent growth patterns. Our results showed that peri‐tumoral mucosal atrophy was identified in 58% of GG, with excellent inter‐rater reliability (*κ* = 0.88, 95% CI: 0.74–1.0). The rationale for the Positive‐Gastric‐GIST‐PG‐CEA criterion relies on local mucosal atrophy with subsequent PG reduction. As PG is exclusively produced by gastric mucosa, this criterion is unlikely to function as a biomarker for non‐gastric GISTs. Whether peri‐tumoral glandular atrophy occurs in non‐gastric GISTs requires further investigation. Should such localized atrophy be confirmed in non‐gastric sites, novel biomarkers could potentially be explored.

The PG and CEA tests are noninvasive, repeatable, radiation‐free, cost‐effective, and highly acceptable methods for screening. Given the widespread clinical application of PGI, PGI/PGII ratio, and CEA, integrating these serum biomarkers into a comprehensive panel can significantly improve GG screening efficiency without additional diagnostic costs. As serum biomarkers, the convenience, repeatability, and radiation‐free nature of serum biomarkers PG and CEA make them more suitable for widespread clinical adoption. Future studies can focus on their role as biomarkers to evaluate the effect of therapy and postoperative surveillance. Small GG are often negative on CT scans and endoscopy. Endoscopic ultrasonography can be considered when subjects meet the Positive‐Gastric‐GIST‐PG‐CEA criteria, especially for subjects with high GG risk, such as those with familial GISTs and those with succinate dehydrogenase complex dysfunction. Although GISTs are relatively rare in the elderly population, these cases often require emergency surgical management [56]. Our 10‐year stratified subgroup analysis revealed that while the sensitivity of this biomarker panel declines with age, it still maintains a sensitivity of 50% in patients aged ≥ 70 years. This noninvasive testing method holds important clinical value for elderly GIST patients, potentially reducing the need for emergency surgery.

Although baseline characteristics showed some imbalance between GG and GC groups, the study cohort remained representative of the general patient population. In the real world, the prevalence of GC in male patients is 2.38 times that of female patients in China. In contrast, while most GIST studies report no significant gender differences, both Norwegian registry data [57] and our cohort found female predominance. These two malignancies demonstrate distinct clinicopathological characteristics, with GC predominantly occurring in the gastric antrum and exhibiting aggressive metastatic potential, whereas GG rarely involves the antral region and displays minimal metastatic tendency. These characteristic differences were consistently observed throughout our dataset.

There were some limitations in this study. First, the retrospective, single‐center design may introduce selection bias, although the follow‐up rate was relatively high (137/199 GG patients completed follow‐up). Second, all PG measurements in this study were performed exclusively using Abbott assay kits, necessitating further validation to determine the generalizability of our criteria to other commercial testing platforms. Third, the assessment of mucosal atrophy was restricted by sample availability, as some histological slides were unavailable or resection GG tumor specimens lacked adequate adjacent mucosa for evaluation. Despite these limitations, our study proposes a novel GG detection strategy with significant clinical implications. Future studies should adopt a prospective, multi‐center design to enhance patient diversity and improve the external validity of the findings. A priori power analysis should be conducted to ensure an adequate sample size, improving the study's ability to detect clinically meaningful differences. Additionally, validating the assessment criteria across multiple PG testing platforms would strengthen robustness and generalizability. Finally, standardizing tissue collection protocols and collaborating closely with pathology departments will help ensure the inclusion of all relevant GG specimens and adjacent mucosa for comprehensive evaluation.

In summary, this study identified that localized glandular atrophy in the peri‐tumoral gastric mucosa represents a characteristic histopathological feature of GG. The Positive‐Gastric‐GIST‐PG‐CEA criteria can effectively distinguish the most common GG from the most common GC. The criteria showed high sensitivities in all GG subgroups. Integrating our criteria into the current PG test scheme of gastric precancerous screening would be beneficial for the detection of GG without additional expense. In fact, more than 90% of GG patients are diagnosed over the age of 40, which is also the age of subjects suitable for Japanese government‐sponsored AG screening by PG.

## Author Contributions

Likun Zhou: study design, data collation, patient follow‐up, data analysis, data interpretation, pathological slides collection, and article writing. Yi Ba: study design, data interpretation, and article writing. Zhiying Gao: data collation, data analysis, data interpretation, pathological slides reading, and article writing. Laizhi Luo: data collation, patients follow‐up, pathological slides collection, and article writing. Yueting Han: laboratory results collection. Yan Sun: pathological slides reading. Yonghong Huang: pathological slides reading. Shixia Li: study design comments. Xingyun Chen: patients follow‐up, pathological slides collection. Huimin Yang and Zhijuan Peng: patients follow‐up. Xinyi Wang: pathological slides collection, data collection. Wei Zhao, Xi Wu, Huan Wu, Jing Bai, and Wu Sun: article writing. All authors commented on previous versions of the manuscript. All authors read and approved the final manuscript.

## Ethics Statement

The ethical approval of this study was approved by the institutional review board of Tianjin Cancer Hospital.

## Consent

The informed consent was waived by the institutional review board of Tianjin Cancer Hospital because this was a retrospective study, and all patients were discharged.

## Conflicts of Interest

The authors declare no conflicts of interest.

## Supporting information


**Table S1:** PG levels of the ten gastric GISTs (GG) patients.
**Table S2:** Additional Characteristics of GG Group.
**Table S3:** Performance Evaluation of Serological Criteria Combined with MLP Model for GG prediction in the internal validation cohort.
**Table S4:** USS scoring of peri‐tumoral mucosa in patients with GG.
**Table S5:** Subgroup Analysis of Sensitivity in GG group.
**Table S6:** Subgroup Analysis of Sensitivities in GG group.

## Data Availability

The data that support the findings of this study are available on request from the corresponding author. The data are not publicly available due to privacy or ethical restrictions.
