# Supplementary material for: Serum Pepsinogen as a Biomarker of Gastrointestinal Stromal Tumors (GIST) in Stomach
Source: Cancer Med. 2025 Sep 3;14(17):e71186. doi: 10.1002/cam4.71186 (PMC12406084; doi:10.1002/cam4.71186)
Supplement: Supplementary file 1 — Table S1: PG levels of the ten gastric GISTs (GG) patients. Table S2: Additional Characteristics of GG Group. Table S3: Performance Evaluation of Serological Criteria Combined with MLP Model for GG prediction in the internal validation cohort. Table S4: USS scoring of peri‐tumoral mucosa in patients with GG. Table S5: Subgroup Analysis of Sensitivity in GG group. Table S6: Subgroup Analysis of Sensitivities in GG group. [file CAM4-14-e71186-s001.docx]

***Title PAGE***

***Serum Pepsinogen as a Biomarker of Gastrointestinal Stromal Tumors (GIST) in Stomach***

Zhiying Gao^1^; Laizhi Luo^2^; Yueting Han^3^; Yan Sun^3^; Yonghong Huang^4^; Shixia Li^3^; Xingyun Chen^3^; Huimin Yang^3,5^; Zhijuan Peng^3,6^; Xinyi Wang^3^; Wei Zhao^7^; Xi Wu^8^; Huan Wu^9^; Jing Bai^10^; Wu Sun^11^; Likun Zhou^3^; and Yi Ba^1,3^

Correspondence: Likun Zhou, Tianjin Medical University Cancer Institute and Hospital, National Clinical Research Center for Cancer, Tianjin's Clinical Research Center for Cancer, Key Laboratory of Cancer Prevention and Therapy, Tianjin, China; e-mail: zhoubaling123@163.com | Yi Ba, MD, PhD, Peking Union Medical College Hospital, Chinese Academy of Medical Sciences & Peking Union Medical College, Beijing, China; e-mail: bayi@tjmuch.com

Affiliations

1. Peking Union Medical College Hospital, Chinese Academy of Medical Sciences & Peking Union Medical College, Beijing, China
2. Guangzhou Medical University, Guangzhou 523808, China
3. Tianjin Medical University Cancer Institute and Hospital, National Clinical Research Center for Cancer, Tianjin's Clinical Research Center for Cancer, Key Laboratory of Cancer Prevention and Therapy, Tianjin, China
4. Institute of Pathology, Qiqihar Medical University, Qiqihar, China
5. Handan Central Hospital, Handan, China
6. People's Hospital of Xiangxi Autonomous Prefecture, Xiangxi, China
7. Gastrointestinal department of Tianjin Medical University General Hospital, Tianjin, China
8. National Cancer Center/National Clinical Research Center for Cancer/Cancer Hospital, Chinese Academy of Medical Sciences and Peking Union Medical College, Beijing, China
9. Ultrasound department of Sun Yat-Sen Memorial Hospital, Sun Yat-Sen University, Guangzhou, China
10. Beijing Friendship Hospital, Capital Medical University, Beijing, China
11. The Comprehensive Cancer Centre of Drum Tower Hospital, Medical School of Nanjing University and Clinical Cancer Institute of Nanjing University, Nanjing, China

**Supporting Information**

**This supplementary file includes:**

1. Supporting Tables S1 to S6

**TABLE S1. PG levels of the ten gastric GISTs (GG) patients.**

| **NO.** | **PGⅠ(ng/mL)** | **PGⅡ(ng/mL)** | **PGⅠ/PGⅡ** | **Using PPIs before PG test** | **Gastric mucosa surrounding GIST** |
| --- | --- | --- | --- | --- | --- |
| **patient 1** | 56.2 | 5.7 | 9.9 | No | Unavailable |
| **patient 2** | 120.6 | 39.4 | 3.1 | No | No surrounding gastric mucosa |
| **patient 3** | 52.8 | 11.2 | 4.7 | No | Atrophy |
| **patient 4** | >200 | 32.9 | >3 | No | Atrophy |
| **patient 5** | 136.2 | 30.8 | 4.4 | Yes | Unavailable |
| **patient 6** | 87.5 | 16.1 | 5.4 | Yes | No surrounding gastric mucosa |
| **patient 7** | 58.6 | 4.8 | 12.2 | Unknown | Unavailable |
| **patient 8** | 68.2 | 19.6 | 3.5 | Unknown | Atrophy |
| **patient 9** | 77.5 | 11 | 7.1 | Unknown | Unavailable |
| **patient 10** | 160.5 | 31.9 | 5 | Unknown | No atrophy |

PG: pepsinogen; PPIs: proton pump inhibitors.

**TABLE S2. Additional Characteristics of GG Group.**

|  |  | **No. of Gastric GISTs patients**  ***N*=100** |
| --- | --- | --- |
| **Initial symptoms-no.** |  |  |
|  | Symptomless | 48 |
|  | Abdominal pain | 15 |
|  | Abdominal distention | 6 |
|  | Other Epigastric discomfort | 17 |
|  | Reflux | 7 |
|  | Accidental found when treating other disease | 4 |
|  | diarrhea | 2 |
|  | GI bleeding | 1 |
| ***Hp* infection-no.** |  |  |
|  | Positive | 19 |
|  | Negative | 12 |
|  | Unknown | 69 |
| **CD117 positive-no.** |  | 95 |
| **Dog-1 positive-no.** |  | 98 |
| **CD34 positive-no.** |  | 98 |
| **C-kit mutation-no.** |  |  |
|  | No mutation | 13 |
|  | Exon 11 mutation | 41 |
|  | Exon 17 mutation | 2 |
|  | Untested-no. | 44 |
| **PDGFR mutation-no.** |  |  |
|  | No mutation | 48 |
|  | Exon 18 mutation | 3 |
|  | Untested | 49 |
| **Lesion number-no.** | 1 | 96 |
|  | 2 | 2 |
|  | 3 | 1 |
|  | Unknown | 1 |

PDGFR denotes Platelet Derived Growth Factor Receptor.

**Table S3. Performance Evaluation of Serological Criteria Combined with MLP Model for GG prediction in the internal validation cohort.**

| Model | AUC (95% CI) | Accuracy (95% CI) | Specificity (95% CI) | Sensitivity (95% CI) | PPV (95% CI) | NPV (95% CI) | *F*_1_-score (95% CI) |
| --- | --- | --- | --- | --- | --- | --- | --- |
| Gender-PGI-PGR-CEA**^*^** | 0.854 (0.829, 0.88) | 0.792 (0.754, 0.83) | 0.867 (0.81, 0.925) | 0.660 (0.551, 0.769) | 0.754 (0.682, 0.826) | 0.820 (0.779, 0.862) | 0.693 (0.624, 0.763) |
| Gender-Positive-Gastric-GIST-PG-CEA**^#^** | 0.804 (0.746, 0.862) | 0.759 (0.713, 0.805) | 0.908 (0.857, 0.959) | 0.500 (0.445, 0.555) | 0.768 (0.662, 0.873) | 0.759 (0.733, 0.786) | 0.603 (0.534, 0.673) |
| Positive-Gastric-GIST-PG-CEA**^#^** | 0.772 (0.709, 0.835) | 0.748 (0.697, 0.799) | 0.787 (0.738, 0.837) | 0.680 (0.599, 0.761) | 0.649 (0.585, 0.713) | 0.811 (0.768, 0.855) | 0.663 (0.594, 0.731) |

*denotes analysis based on continuous variables; # denotes analysis based on binary variables.

**TABLE S4. USS scoring of peri-tumoral mucosa in patients with GG.**

| NO. | Gender | Age | PGI (ng/mL) | PGII (ng/mL) | PGI/PGII ratio | CEA (µg/L) | USS score |
| --- | --- | --- | --- | --- | --- | --- | --- |
| patient 1 | Female | 65 | 81.9 | 17.5 | 4.7 | 0.99 | 3 |
| patient 2 | Male | 64 | 83.4 | 14 | 6 | 1.61 | 3 |
| patient 3 | Female | 70 | 58.5 | 6.3 | 9.3 | 1.91 | 3 |
| patient 4 | Female | 61 | 100.1 | 23.8 | 4.2 | 1.31 | 3 |
| patient 5 | Female | 43 | 44.2 | 9.1 | 4.9 | 0.34 | 3 |
| patient 6 | Female | 82 | 12.4 | 8.9 | 1.4 | 2.12 | 2 |
| patient 7 | Female | 56 | 49.8 | 7 | 7.1 | 1.05 | 2 |
| patient 8 | Female | 68 | 79.5 | 17.5 | 4.5 | 3.75 | 2 |
| patient 9 | Female | 50 | 41.4 | 4.5 | 9.2 | 0.89 | 2 |
| patient 10 | Female | 55 | 23.3 | 16.7 | 1.4 | 2.05 | 2 |
| patient 11 | Male | 65 | 32.9 | 4.8 | 6.9 | 1.29 | 2 |
| patient 12 | Female | 58 | 41.3 | 4.9 | 8.4 | 2.21 | 2 |
| patient 13 | Male | 55 | 27.4 | 4.1 | 6.7 | 1.74 | 2 |
| patient 14 | Male | 62 | 59.1 | 6.9 | 8.6 | 0.68 | 2 |
| patient 15 | Female | 74 | 72.6 | 16.3 | 4.5 | 2.94 | 1 |
| patient 16 | Female | 66 | 5.5 | 11.5 | 0.5 | 0.81 | 1 |
| patient 17 | Male | 67 | 66.1 | 9.1 | 7.3 | 1.98 | 1 |
| patient 18 | Male | 52 | 59.2 | 7.3 | 8.1 | 3.43 | 1 |
| patient 19 | Male | 72 | 57.6 | 11.4 | 5.1 | 1.43 | 1 |
| patient 20 | Female | 61 | 35.7 | 4.1 | 8.7 | 1.67 | 1 |
| patient 21 | Female | 68 | 4.5 | 12.2 | 0.4 | 1.49 | 1 |
| patient 22 | Female | 81 | 157.2 | 26.5 | 5.9 | 2.37 | 1 |
| patient 23 | Male | 34 | 48.5 | 9.4 | 5.2 | 1.04 | 1 |
| patient 24 | Male | 60 | 45.2 | 6.9 | 6.6 | 3.54 | 1 |
| patient 25 | Female | 56 | 82.6 | 17.6 | 4.7 | 1.04 | 1 |
| patient 26 | Male | 67 | 102.3 | 21.1 | 4.9 | 1.79 | 1 |
| patient 27 | Male | 64 | 70.8 | 9.3 | 7.6 | 4.91 | 1 |
| patient 28 | Female | 73 | 44.4 | 10.3 | 4.3 | 4.14 | 1 |
| patient 29 | Female | 40 | 20.1 | 3 | 6.7 | 0.71 | 1 |
| patient 30 | Male | 52 | 72.6 | 8.2 | 8.9 | 3.80 | 0 |
| patient 31 | Female | 62 | 163.7 | 38.2 | 4.3 | 1.01 | 0 |
| patient 32 | Male | 42 | 39.5 | 2.4 | 16.5 | 1.64 | 0 |
| patient 33 | Female | 48 | 66.3 | 10.4 | 6.4 | 1.18 | 0 |
| patient 34 | Female | 43 | 41.3 | 5.7 | 7.3 | 0.77 | 0 |
| patient 35 | Female | 59 | 38.7 | 6.5 | 6 | 3.96 | 0 |
| patient 36 | Male | 63 | 158.5 | 35 | 4.5 | 2.78 | 0 |
| patient 37 | Female | 65 | 38.9 | 6.2 | 6.3 | 1.77 | 0 |
| patient 38 | Female | 54 | 45.3 | 5.6 | 8.1 | 1.46 | 0 |
| patient 39 | Female | 63 | 65.9 | 6.7 | 9.8 | 2.24 | 0 |
| patient 40 | Female | 72 | 58.1 | 7.5 | 7.8 | 4.58 | 0 |
| patient 41 | Male | 46 | 107.9 | 24.4 | 4.4 | 0.82 | 0 |
| patient 42 | Female | 56 | 46.1 | 13.6 | 3.4 | 2.53 | 0 |
| patient 43 | Female | 59 | 51.8 | 7.4 | 7 | 2.59 | 0 |
| patient 44 | Female | 54 | 36.9 | 5.5 | 6.7 | 4.12 | 0 |
| patient 45 | Female | 49 | 42.7 | 5.1 | 8.4 | 2.24 | 0 |
| patient 46 | Female | 45 | 55.6 | 9.7 | 5.7 | 0.57 | 0 |
| patient 47 | Male | 46 | 36.7 | 6.5 | 5.7 | 2.98 | 0 |
| patient 48 | Male | 36 | 136.5 | 23.3 | 5.9 | 1.56 | 0 |
| patient 49 | Female | 44 | 41.8 | 5.6 | 7.5 | 1.45 | 0 |
| patient 50 | Female | 59 | 41.5 | 5 | 8.3 | 1.59 | 0 |

**Table S5. Subgroup Analysis of Sensitivity in GG group.**

|  |  | No. of patients | Positive-Gastric-GIST-PG-CEA |
| --- | --- | --- | --- |
| Tumor location-no. (%) |  |  |  |
|  | EGJ, cardia and fundus | 43 | 30 (70) |
|  | Corpus | 47 | 31 (66) |
|  | Corner and antrum | 9 | 6 (67) |
| Tumor size-no. (%) |  |  |  |
|  | ≤1cm | 11 | 10 (91) |
|  | ＞1≤2cm | 6 | 4 (67) |
|  | ＞2≤5cm | 33 | 22 (67) |
|  | ＞5≤10cm | 35 | 21 (60) |
|  | ＞10cm | 14 | 11 (79) |
| Recurrent risk-no. (%) |  |  |  |
|  | None risk | 17 | 14 (82) |
|  | Very low risk | 26 | 15 (58) |
|  | Low risk | 25 | 17 (68) |
|  | Moderate risk | 9 | 9 (100) |
|  | High risk | 15 | 8 (53) |
| Lesion growth pattern |  |  |  |
|  | Exophytic mass | 29 | 20 (69) |
|  | Endophytic mass | 14 | 9 (64) |
| Initial symptoms-no. (%) |  |  |  |
|  | Symptomless | 48 | 29 (60) |
|  | Abdominal pain | 15 | 8 (53) |
|  | Abdominal distention | 6 | 6 (100) |
|  | Other epigastric discomfort | 17 | 12 (71) |
|  | Reflux | 7 | 7 (100) |
|  | Accidentally found when treating other diseases | 4 | 4 (100) |
|  | Diarrhea | 2 | 1 (50) |
|  | Gastrointestinal bleeding | 1 | 1 (100) |
| Sex-no. (%) |  |  |  |
|  | Male | 32 | 20 (63) |
|  | Female | 68 | 48 (71) |
| Age-no. (%) |  |  |  |
|  | ≤39 | 5 | 4 (80) |
|  | ＞39≤49cm | 17 | 16 (94) |
|  | ＞49≤59cm | 28 | 20 (71) |
|  | ＞59≤69cm | 36 | 21 (58) |
|  | ＞69 | 14 | 7 (50) |
| *Hp* infection status-no. (%) |  |  |  |
|  | *Hp* negative | 19 | 15 (79) |
|  | *Hp* positive | 12 | 5 (42) |
|  | *Hp* infection unknown | 69 | 48 (70) |

EGJ: esophagogastric junction; *Hp*: Helicobacter pylori; Positive-Gastric-GIST-PG-CEA: serum PGI < 70 ng/mL, PGI/PGII ratio ≥ 3.0, and CEA ≤ 5 µg/L.

**TABLE S6. Subgroup Analysis of Sensitivities in GG group.**

|  |  | **No. of patients** | **Positive-Gastric-GIST-PG-CEA** |
| --- | --- | --- | --- |
| **Tumor location-no. (%)** |  |  |  |
|  | EGJ and fundus | 36 | 7(19) |
|  | Corpus | 59 | 15(25) |
|  | Corner and antrum | 61 | 11(18) |
|  | Whole stomach | 6 | 2(33) |
|  | Location unknown | 12 | 3(25) |
| **Tumor size-no. (%)** |  |  |  |
|  | ≤1cm | 2 | 1(50) |
|  | ＞1≤2cm | 13 | 5(38) |
|  | ＞2≤5cm | 61 | 8(13) |
|  | ＞5≤10cm | 43 | 13(30) |
|  | ＞10cm | 5 | 2(40) |
|  | Size unknown | 50 | 9(18) |
| **Cancer differentiation-no. (%)** |  |  |  |
|  | Highly differentiated adenocarcinoma | 1 | 0(0) |
|  | Moderately differentiated adenocarcinoma | 21 | 6(29) |
|  | Poorly differentiated adenocarcinoma | 95 | 20(21) |
|  | Signet-ring cell carcinoma | 36 | 10(28) |
|  | Differentiation unknown | 21 | 2(10) |
| **Stage-no. (%)** |  |  |  |
|  | Stage 1 | 24 | 9(38) |
|  | Stage 2 | 24 | 8(33) |
|  | Stage 3 | 48 | 10(21) |
|  | Stage 4 | 56 | 6(11) |
|  | Stage unknown | 22 | 5(23) |
| **Sex-no. (%)** |  |  |  |
|  | Male | 126 | 30(24) |
|  | Female | 48 | 8(17) |
| **Age-no. (%)** |  |  |  |
|  | ≤39 | 7 | 1(14) |
|  | ＞39≤49cm | 20 | 5(25) |
|  | ＞49≤59cm | 43 | 11(26) |
|  | ＞59≤69cm | 68 | 12(18) |
|  | ＞69 | 36 | 9(25) |

PG: Pepsinogen; EGJ: esophagogastric junction; Positive-Gastric-GIST-PG-CEA: serum PGI < 70 ng/mL, PGI/PGII ratio ≥ 3.0, and CEA ≤ 5 µg/L.
